# Physical Activity and Health Beliefs among Saudi Women

**DOI:** 10.1155/2012/642187

**Published:** 2012-02-22

**Authors:** Einas S. Al-Eisa, Hana I. Al-Sobayel

**Affiliations:** ^1^College of Applied Medical Sciences, King Saud University, P.O. Box 1684, Riyadh 11441, Saudi Arabia; ^2^College of Applied Medical Sciences, King Saud University, P.O. Box 6941, Riyadh 11452, Saudi Arabia

## Abstract

*Background*. Physical activity (PA) is associated with health benefits and disease prevention and is often prescribed in managing many health conditions. Understanding the cultural influences is relevant in order to effectively promote PA. The objective of this study was to assess the level of PA among Saudi women, measured by daily step count, and the association between PA and health beliefs. *Methods*. A total of 161 eligible participants were asked to complete two questionnaires to assess health beliefs: Health Locus of Control (HLC) and Self-Efficacy Assessment Scale. Each participant was given a pedometer and a diary to record their daily PA for two weeks. *Results*. One hundred and five participants completed the two weeks pedometer data (mean age 26.3 ± 7.1 years, BMI 25 ± 4.2 kg/m^2^). The average pedometer score over two weeks was 5114 ± 2213 steps. Step count had strong correlation with self-efficacy (*r*_*s*_ = 0.75), mild correlation with internal HLC (*r*_*s*_ = 0.42), and mild negative correlation with external HLC (*r*_*s*_ = −0.35). *Conclusion*. The study demonstrates high level of inactivity among Saudi females in reference to the international recommendation for minimum activity. The data also reveal an association between PA and health beliefs. Ultimately, such information can be used to design gender- and culture-sensitive interventions that could enhance adherence to PA.

## 1. Introduction

It is widely reported that regular physical activity reduces the risk of a number of medical conditions, and contributes to personal wellbeing [1]. The World Health Organization (WHO) regards physical inactivity as one of the leading causes of death and disability [2], and a leading cause of noncommunicable chronic conditions, such as hypertension, diabetes, and obesity [3].

As a result of this, clinical practitioners often prescribe physical activity as an essential component for the management of many health conditions [4]. However, medical advice alone has not been effective in promoting physical activity [5], resulting in extensive research on the means to promote physical activity. Despite this, there is no universally accepted method due to the multiple factors that influence physical activity.

In order to facilitate the promotion of physical activity, an understanding of social and cultural influences is of paramount importance. It is well known and generally accepted that different cultures have different health outcomes influenced by different beliefs [6]. Sociodemographic factors have also been examined in relation to health promoting behaviors, but inconsistent results were reported [7–9]. Overall, there is sufficient evidence in the literature to show that the level of physical activity among societies is influenced by health beliefs, psychosocial status, and self-efficacy [9].

Significant increase in the level of physical inactivity among the Saudi population has been recently reported, predisposing them to health problems [10, 11]. So far, the majority of studies conducted on the Saudi society have focused on either the male population, or children and adolescents [12, 13], however, the prevalence of sedentary lifestyle-related obesity has been escalating among Saudi females [14]. This demonstrates a growing need to understand how social restraints imposed upon Saudi women affect women's health and their response to current treatment methods.

The literature suggests a specific gender-based consideration when making recommendations to promote physical activity [15]. This means that intervention and motivation programs should be customized to suit the needs of the individual, with gender as a primary consideration.

In Saudi Arabia, women are prohibited from driving and require a guardian for commuting. Such cultural factors faced by Saudi females could prohibit or limit exercise activities, thereby increasing the prevalence of physical inactivity among such population [10]. For this reason, it is deemed necessary to study factors affecting physical activity in the Saudi culture in order to design appropriate interventions to improve it. To our knowledge, the identification of a base-line activity level of Saudi females has not been previously established.

There are number of methods reported in the literature to measure physical activity [16]. The most frequent of these is the use of self-report diaries or retrospective recall questionnaires, yet the subjective nature of such tools reduces their validity [17]. Recently, the use of pedometers has been considered an objective measure of physical activity [18]. Validity of the pedometers in measuring step count has been widely established [19, 20].

The use of pedometers to assess physical activity has been justified by the fact that walking is the most widely advocated form of physical activity. Walking is suggested as the mode of activity most likely to increase physical activity at a population level [21], particularly for sedentary adults [22]. Walking behavior has received recent attention based on its physical and psychological health benefits [23, 24], and its ease to physically perform and low cost in comparison to other forms of physical activity [25, 26].

This paper seeks to quantify the level of physical activity among Saudi females. The secondary objective of the study is to investigate the psychosocial factors associated with level of physical activity among Saudi females. Given the apparent interaction between culture, beliefs, and health in the literature, emphasis will be placed on investigation of the relationship between the level of physical activity and health beliefs.

## 2. Methods

### 2.1. Participants

Participants were recruited through advertisement using posters and flyers, university and hospital newsletters, through primary health care centers, and announcements in female health clubs and community centers in Riyadh city. Literate Saudi females between the ages of 18 and 45 years old were targeted. Pregnant volunteers were excluded, as well as those with history of fracture or surgery to the back, pelvis, or lower limb. Participation in the study was also prohibited by eating disorders, conditions affecting cognitive function, or conditions affecting the ability to walk.

### 2.2. Instrumentation

#### 2.2.1. Measurement of Physical Activity

To assess the level of physical activity, step counts were measured by the *Omron HJ-152K-E pedometer*, which has been reported to have acceptable validity and reliability [27].

#### 2.2.2. Measurement of Health Beliefs

To assess health beliefs, the Arabic versions of two well-established and validated questionnaires were used.

**Multidimensional Health Locus of Control (HLC)**

It contains three 6-item subscales: internality; powerful others externality; chance externality. Each item scored from 1 (strongly disagree) to 6 (strongly agree) for the externally worded items and reverse scored for the internally worded items [28]. Those who have confidence that whatever happens to them is substantially within their domain of influence are said to have a predominantly internal locus of control, while those who believe that they are influenced by external forces are considered to have an external locus of control [29]. The Arabic version of the HLC was constructed and validated by Badr and Moody [6].

**Self-Efficacy Assessment Scale**

It comprises of 10 items scored on a 4-point Likert scale, to examine beliefs about ability to cope with a large variety of stressors [30]. This scale measures the belief people have in their own abilities to perform the desired behaviors in various situations [31] and was validated in different languages including Arabic [30].

### 2.3. Procedure

Phone interviews were conducted to screen all interested volunteers to ensure eligibility to the study. Consent was sought and obtained from each eligible participant. Ethical approval for the study was granted by the University Institutional Review Board.

On the first visit, baseline assessment was conducted and participants demographics (age, occupation, education, marital status, residential area) and anthropometric measures (weight, height, BMI) were taken. At this baseline assessment, participants were asked to complete the HLC Questionnaire and the Self-Efficacy Assessment Scale.

Each participant was given a pedometer to wear daily for 2 weeks. A two-week data collection period was selected to attain accurate information regarding the usual physical activity pattern. Also, the motivational effect of the pedometer use, and the associated bias were eliminated by the prolonged period. Participants were asked to record the pedometer reading (number of steps) in a diary at the end of each day. The diaries provide a log of daily step count. All participants were encouraged to maintain their typical level of walking to obtain a baseline indicator of their activity.

### 2.4. Data Analysis

A cross-sectional research design was used. Descriptive statistics were computed to describe the participants' demographics and anthropometric data.

The analysis strategy was based on themes that emerged from the literature. Since beliefs are considered multidimensional with a poorly understood causal structure, a univariate analysis approach is often desirable over a multivariate analysis [26]. Hence, a univariate correlational analysis was conducted to examine the relationship between the level of physical activity and measures of health beliefs. Walking behavior was considered the critical dependent variable. Specifically, nonparametric correlations were made between the step count obtained by the pedometers with the participants' self-reports in the two questionnaires. Strength of association was assessed using Glantz classification [32].

## 3. Results

A total of 320 volunteers were initially screened, of which 161 volunteers were recruited based on the inclusion criteria. Demographic data are presented in Table 1. One hundred and five subjects completed the two weeks step-count data (mean age = 26.3 ± 7.1 years; mean BMI: 25 ± 4.2 kg/m^2^). All participants had a minimum education of high school degree, and 31.4% had a college degree (Table 2). The majority of the sample was university students at the time of the study (71%), and the remaining were workers (29%). The average number of steps taken daily by our sample was 5114 (±2213). Step count did not correlate with the participants' demographics, social status, or residential location.

The outcome measures scores are presented in Table 3. Overall, participants had higher internal HLC than external HLC, with particularly lower chance HLC. Participants were found to have moderate to high self-efficacy. Step count had strong correlation with self-efficacy (*r*_*s*_ = 0.75, *P* = 0.03), mild correlation with internal HLC (*r*_*s*_ = 0.42, *P* = 0.02), and mild negative correlation with external HLC (*r*_*s*_ = −0.35, *P* = 0.03).

## 4. Discussion

This study was derived on the premise that factors such as gender, age, occupational stressors, socioeconomic status are likely to influence the outcome of health [9]. The results support the view that health behaviors are associated with health beliefs, particularly HLC (beliefs about what controls one's health) [33], and self-efficacy (beliefs about one's ability to cope with stressors) [30].

The notion of perceived locus of control is the most widely known of the psychological constructs associated with health beliefs [34]. Health locus of control has been extensively studied in relation to health behaviors [35]. These results support previous work suggesting that higher internal HLC is positively associated with higher performance of regular physical activity, while external HLC is negatively associated with it [36]. Internal scoring people have a greater tendency to attribute life outcomes to personal characteristics relating to ability, effort, and personal power of control [37]. On the other hand, people who score higher in external locus of control are more likely to attribute successes and failures in life to factors such as fate, luck, and chance [37, 38]. It seems natural that those with high internal locus of control assume control over their health and thereby tend to have a high level of physical activity, and that the opposite is true for people with high external locus of control.

Self-efficacy is another concept that is also widely researched in health promotion [39, 40]. The current data confirmed the positive role of efficacy beliefs in initiating and maintaining a regular program of physical exercise [41]. The novelty in this study lies in challenging the empirical assumption that Arabs have higher external and lower internal HLC, and thus fewer health-promoting behaviors [37]. This sample of Saudi young educated females had higher internal HLC suggesting that they assume more responsibility for their actions. Previous evidence suggests that Arabs tend to perceive forces outside the individual as causing illness, thus expressing higher external HLC [42]. According to Cohen and Azaiza [37], these perceptions are incorporated in the cultural belief system and are thus not always related to level of education. In contrast to this view, other scholars note that the Islamic religion stresses that individuals take personal responsibility for their health and that it encourages active health-promoting behaviors [43].

One of the key findings of this study is the low number of steps taken by Saudi females, which is substantially lower than the widely promoted target of 10,000 steps per day required to attain health-related benefits [20]. The mean number of steps of this sample would place them in the “low active” category (5000–7499 steps/day) of Tudor-Locke and Bassett [20]. This low level of physical activity has never been reported in similar age groups, and, instead, mimics the level of activity in adults 65 years of age or older [44–47]. Other reports of physical inactivity among men and women in the Gulf Cooperation Council, including Saudi Arabia, also showed low levels of sufficient physical activity, more prominent in women compared to men [48]. Estimates were 39.0–42.1% for men and 26.3–28.4% for women, based on only two nationally representative samples from Saudi Arabia and Kuwait. However, those reports were based on data collected using self-report questionnaires on physical activity only, and as such cannot be conclusively verified.

These findings could be analyzed in the context of the Saudi social system and the role of women in this system. Conservative social norms defining the roles for males and females in Muslim countries influence the context in which they can be physically active and reduce potential weight gain [48]. In Saudi Arabia, women have restrictions to movement outside their homes and limited opportunities to attend health centers [49]. In addition, the hot climate, high dependency on automobiles, as well as the employment of domestic helpers, seem to contribute to low levels of activity in daily life [48].

Data from the WHO (2009) also showed that physical inactivity is globally more prevalent among girls and women than their male counterparts [50]. Many factors hinder the participation of women in physical activity and their access to health care, including lower income for women, required agreement from a senior member of the household to engage in physical activity, having a greater workload in the home and care-giving roles, limited mobility, and cultural restrictions [50].

The social structure in Saudi Arabia tends to remain male-dominated, collectivistic, and patriarchal, with great emphasis on family values and group cohesiveness. Consequently, women who grow up in this kind of society may develop a lower internal sense of control and lower confidence level. This may carry even broader implications, since women will not play their important role in encouraging health behaviors in the family setting and on the community level [51]. This pattern seems to be beginning to reverse itself, since the Saudi society is undergoing a major modernization process in health and other services, resulting from higher urbanization, more education, and more women working outside the home. This was accompanied by a government reform in favor of women and recent legislation to empower women to play their role in the development of the country.

In light of the above discussion, this study demonstrates that there is an association between internality and the likelihood of making healthy choices. Success in adopting and maintaining regular exercise depends largely on the individual's self-regulatory efficacy [52, 53]. Clinicians may need to operationalize these findings by designing strategies and messages to directly promote these specific significant constructs in enhancing physical activity.

Clinicians are also advised to remember that behavior change is a slow process which requires constant attention [54]. However, it is possible to predict health-related behaviors: people with a more internal HLC generally adhere more closely to health regimens, while more externally oriented individuals are less likely to engage in health-protective behaviors [38].

This study needs to be interpreted within the context of its limitations. First, the sampling frame of Riyadh limits the generalizability of the findings to other regions. Other limitations of the study include the high attrition, the homogeneous self-selected sample, and the fact that the study was run in an academic setting.

## 5. Conclusion

It is important to understand the factors and personal characteristics that affect the perseverance of health-promoting behaviors, in order to construct effective interventions. This study provides a baseline assessment of physical activity among Saudi women. The data showed a correlation between physical activity and health beliefs. Practitioners should devote attention and resources to empowering women to take responsibility for promoting personal and family health. This work can contribute to the development of physical activity interventions that are relevant to the Saudi society.

## Figures and Tables

**Table 1:** Participants' demographics and step count (*n* = 105).

Continuous variables	Minimum	Maximum	Mean ± SD
Age (years)	19	44	26.3 (±7.1)
Weight (Kg)	37.4	131.2	73.2 (±16.4)
BMI (kg/m^2^)	15.4	45	25 (±6.2)
Step count (steps)	575	12630	5114 (±2213)

**Table 2:** Social status, education level, and residential area (*n* = 105).

Categorical variables	Frequency *n* (%)
Social status	
Single	69 (65.7%)
Married	21 (20.0%)
Other	15 (14.3%)
Education level	
High school	72 (68.6%)
Bachelor	28 (26.6%)
Diploma	5 (4.8%)
Residential area	
North	21 (20.0%)
South	19 (18.1%)
East	37 (35.2%)
West	17 (16.2%)
Center	11 (10.5%)

**Table 3:** Outcome measures scores (*n* = 105).

	Mean (SD)	Range	Maximum score
Self-efficacy	27.3 (4.9)	15–39	40
Internal HLC	26.8 (4.1)	17–36	36
Chance HLC	18.5 (4.7)	6–29	36
Powerful other HLC	22.3 (4.9)	9–34	36

HLC: Health Locus of Control.
